# Correction to: Containing pandemics through targeted testing of households

**DOI:** 10.1186/s12879-021-06319-w

**Published:** 2021-07-06

**Authors:** André Voigt, Nikolay Martyushenko, Emil Karlsen, Martina Hall, Kristen Nyhamar, Stig William Omholt, Eivind Almaas

**Affiliations:** 1 Department of Biotechnology and Food Science, NTNU - Norwegian University of Science and Technology, Trondheim, Norway; 2 Department of Circulation and Medical Imaging, NTNU - Norwegian University of Science and Technology, Trondheim, Norway; 3 K.G. Jebsen Center for Genetic Epidemiology, NTNU - Norwegian University of Science and Technology, Trondheim, Norway

**Correction to: BMC Infectious Diseases 21, 548 (2021)**

**https://doi.org/10.1186/s12879-021-06256-8**

Following publication of the original article [1], it was noted that due to a typesetting error the figure legends were paired incorrectly.

The caption of Fig. 1 belongs to Fig. 2. And the caption of Fig. 2 belongs to Fig. 1.

**Fig. 1:**
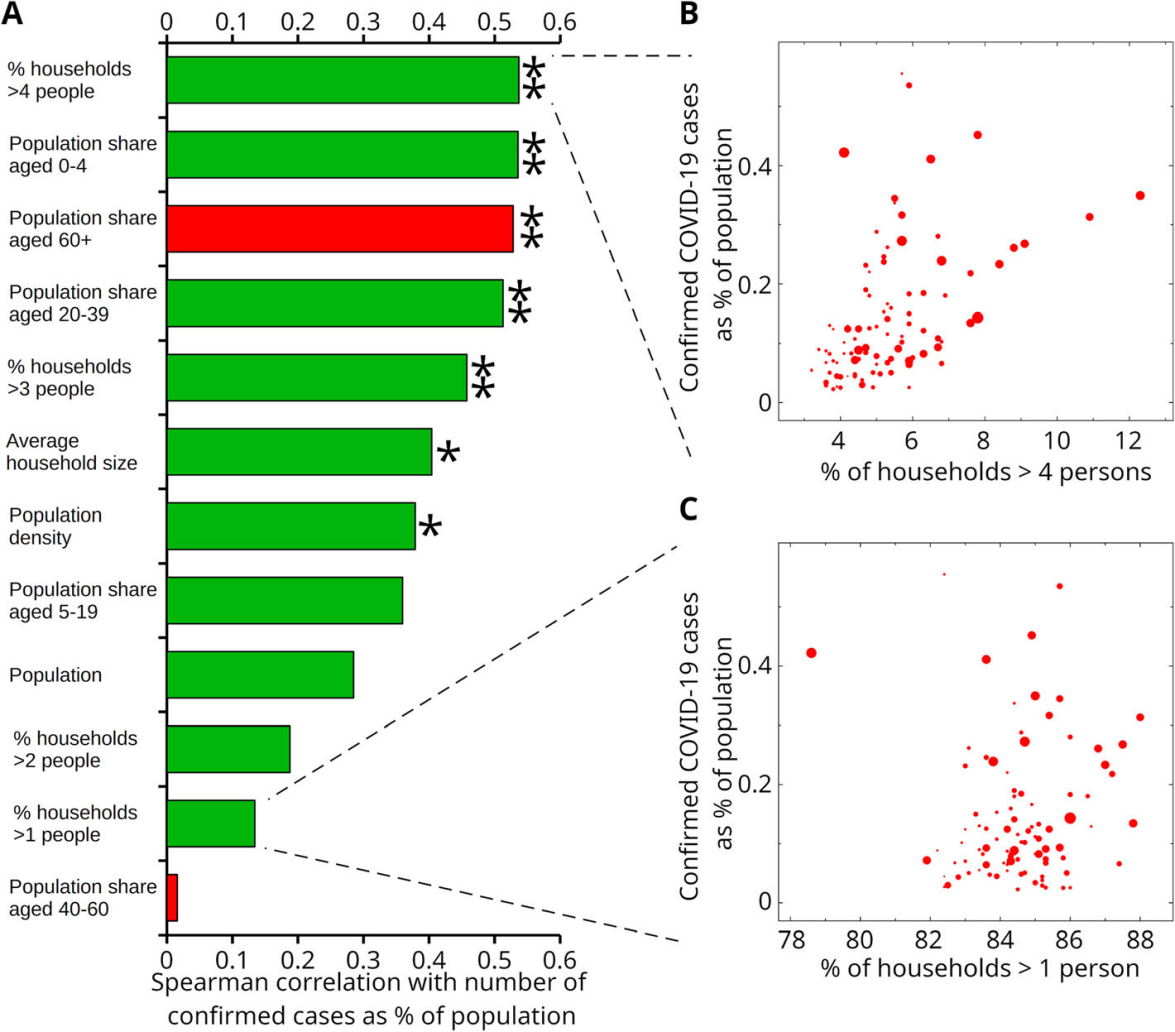
Demographic parameters associated with COVID-19 spread in France. **A** Correlation histogram (positive, green; negative, red) shows larger household sizes significantly correlated with levels of COVID-19 hospitalizations. Single (double) star indicates Bonferroni-corrected significance *P* < 0.01 (*P* < 0.001). Panels **B** and **C** show scatter plots of confirmed cases (as percentage of population) for the 96 departments of European France as function of the percentage of households larger than four persons and one, respectively. Size of markers is proportional to the population of each department

**Fig. 2:**
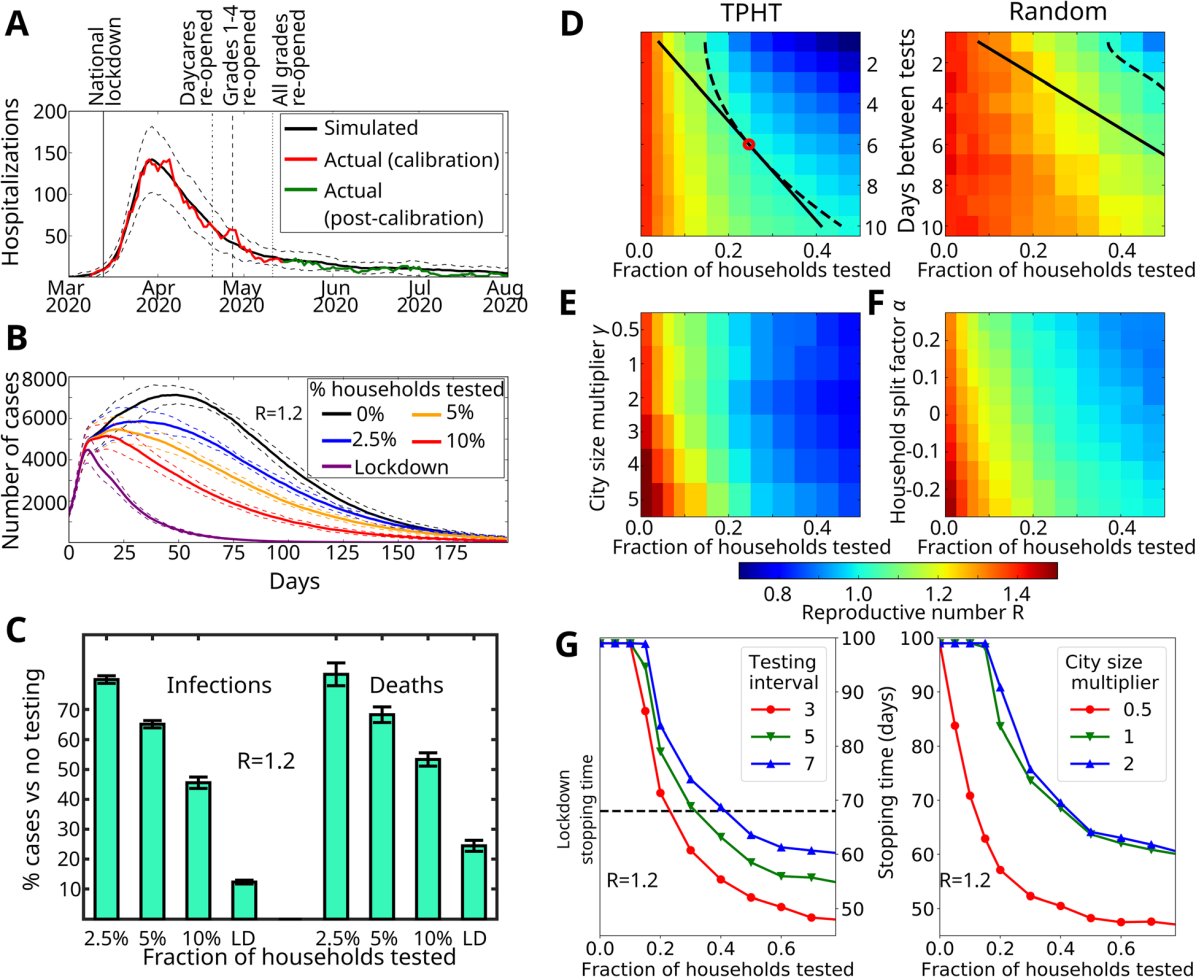
Simulation results and effectiveness of targeted pooled household testing (TPHT) on COVID-19. **A** Fitting the model to Oslo hospitalization data. We plot the mean predicted number of hospitalizations (black) and confidence interval (2σ, dashed). Actual Oslo hospitalizations (red) were used as calibration (until May 15th). Hospitalization data May 15th- August 30th (green) were not used to determine model parameters. **B** Effect of TPHT in response to a sudden rise in cases (reaching 1000 symptomatic individuals), assuming general infectivity parameters similar to those of Oslo in late May 2020 but with 75% increased infectivity of random contacts giving R = 1.2. **C** Predicted number of deaths and infections for different TPHT testing fractions corresponding to panel **B**, relative to no testing. Panels **D**-**F** use same parameters as panel **B**, except with a 113% increased infectivity of random contacts giving R = 1.4. **D** Effect of test frequency and fraction on R, for TPHT (left) and random pooled household testing (right). Dashed and solid lines indicate isoclines for R = 1 and constant test density, respectively. Optimal point is marked with red circle. **E** Response of weekly TPHT to varying city size. We scale the population of the baseline Oslo model (γ = 1) to generate larger (γ > 1) or smaller networks with household, school, daycare, workplace and nursing home size-distributions unchanged. **F** Response of weekly TPHT to changes in distribution of household size, relative to the baseline Oslo model (α = 0). For α > 0, a portion of households are each split into a new pair, yielding a smaller average household size than the baseline. For α < 0, a portion of the households are pairwise merged, yielding a larger average household size than the baseline. Household, school, daycare, workplace and nursing home size-distributions are kept unchanged. **G** COVID-19 stopping time (number of days until symptomatic cases are reduced by 75%) in response to changing days between tests (left) and fraction of weekly TPHT tests (right). Stopping times longer than 100 days are truncated

The correct figures and captions have been included in this correction, and the original article has been corrected.
